# Recent advances in *Brucella abortus* vaccines

**DOI:** 10.1186/s13567-015-0199-7

**Published:** 2015-07-08

**Authors:** Elaine MS Dorneles, Nammalwar Sriranganathan, Andrey P. Lage

**Affiliations:** Departamento de Medicina Veterinária Preventiva, Escola de Veterinária, Laboratório de Bacteriologia Aplicada, Universidade Federal de Minas Gerais, Belo Horizonte, MG Brazil; Department of Biomedical Sciences and Pathobiology, Center for Molecular Medicine and Infectious Diseases, Regional College of Veterinary Medicine, Virginia Polytechnic Institute and State University, Blacksburg, VA USA

## Abstract

*Brucella abortus* vaccines play a central role in bovine brucellosis control/eradication programs and have been successfully used worldwide for decades. Strain 19 and RB51 are the approved *B. abortus* vaccines strains most commonly used to protect cattle against infection and abortion. However, due to some drawbacks shown by these vaccines much effort has been undertaken for the development of new vaccines, safer and more effective, that could also be used in other susceptible species of animals. In this paper, we present a review of the main aspects of the vaccines that have been used in the brucellosis control over the years and the current research advances in the development of new *B. abortus* vaccines.

## Table of contents

1. Introduction
2. Vaccines, vaccination and their use in brucellosis control and eradication programs
3. S19 vaccine
4. RB51 vaccine strain
5. 45/20 vaccine
6. SR82 vaccine strain
7. Vaccination with recombinant genes, proteins, vectors and *B. abortus* recombinant mutants
  7.1. DNA vaccines
  7.2. Subunit vaccines
  7.3. Vector vaccines
  7.4. *B. abortus* recombinant mutants
8. Other *B. abortus* potential vaccines
9. Conclusions
10. Abbreviations
11. Competing interests
12. Authors’ contributions
13. Acknowledgements
14. References

## 1. Introduction

*B. abortus* is the main causative agent of brucellosis in cattle, causing abortion and infertility in adult animals [1]. Bovine brucellosis is a worldwide zoonotic disease, endemic in some regions of the world such as Latin America, Middle East, Africa and Asia [1] and responsible for large economic losses due to animal and human health problems.

Due to public health importance of brucellosis and the damage that it causes to the livestock industry, much effort has been expended to control and eradicate the disease in cattle. The development of an efficacious vaccine for brucellosis control/eradication has been a challenge for scientists for many years. Despite enormous advances and the development of *B. abortus* S19 and RB51 vaccines, the search for improved vaccines has never ends. Although the available vaccines are effective in controlling brucellosis, they have numerous drawbacks, such as interference with diagnostic tests, pathogenicity for humans, potential to cause abortion in pregnant animals, among others. In this paper, we present a review of the main aspects of the vaccines that have been used in the bovine brucellosis control and eradication over the years and some of the current advances in the research for a new *B. abortus* vaccine.

## 2. Vaccines, vaccination and their use in brucellosis control and eradication programs

According to Schurig et al. [2] and Ko and Splitter [3], an ideal vaccine against brucellosis should possess the following characteristics: (i) be live and able to provide a strong type 1 T helper immune response (Th1); (ii) do not induce antibodies that interfere with the serological tests employed in the diagnosis of infected cattle, regardless of route, dose of administration, age or sex of the animals; (iii) be attenuated and do not cause disease or persistent infection in immunized animals nor be pathogenic for humans; (iv) be able to induce a strong and long-lasting protection against systemic and uterine infection, besides preventing abortion, even in pregnant animals inoculated with a single dose; (v) do not lead to seroconversion on revaccination; (vi) be stable and do not revert virulence in vivo nor in vitro; and (vii) be inexpensive, easy to produce and to administer.

Even though we still do not have an ideal vaccine, vaccination with available vaccine strains remains the most successful method for the prevention and control of brucellosis in cattle, being a critical component of most brucellosis control and eradication programs throughout the world [4]. Numerous countries have adopted control measures against bovine brucellosis in order to reduce the prevalence or eradicate the disease from domestic livestock, in an effort to prevent transmission to humans and mitigate economic losses. Vaccination of female calves is the central point of any brucellosis control program, since it has performed well in the reduction of disease prevalence, therefore useful at the disease control stage. Considering that vaccination alone is not enough to control and eradicate the disease, it should be associated with continuous elimination of infected animals, as they are the source of new infections. Thus, besides vaccination, most bovine brucellosis eradicate programs also include test–and–slaughter policies, surveillance and hygiene measures [4].

The aim of vaccination is the reduction of susceptibly individuals in the population and the success of any vaccination program depends mainly on the effectiveness of the vaccine used and its coverage in the target population. Vaccines against brucellosis have been evaluated with respect to their potency by three different approaches: (i) testing in laboratory animals or (ii) testing in natural hosts experimentally challenged and (iii) testing under natural conditions [5]. Of these, test in natural hosts shows more significant response and is the only one able to measure the efficacy of *B. abortus* vaccines [6,7]. In experimental studies of vaccine efficacy, vaccinated and non-vaccinated controls will receive a known infectious dose of a virulent *B. abortus* strain at the most susceptible period (mid-gestation), and the protection is measure by the ability of the vaccine in preventing abortion [4]. However, it is important to emphasize that the experimentally obtained effectiveness may differ from field efficacy that can be influenced by other factors, such as nutrition, environmental stress, age at vaccination, vaccination management or immunological status [4]. Besides, the above three classical methodologies, *B. abortus* vaccines could also be assessed by measuring the immune responses and determination of correlates of protection by mathematical modeling. The identification of protection markers can be an useful approach to screen vaccine candidates whether validated by vaccine potency tests [8]. Since experiments involving challenge of pregnant cattle are very expensive, time-consuming and requires large animal biosafety level 3 facilities, the rational way for the future of *B. abortus* vaccines testing and development could be the characterization and identification of the correlates of protection.

Another important aspect related to the success of brucellosis control programs is the quality of the vaccine used. Despite the cost of the vaccine being just one fraction of the total cost of a control program, its quality will affect directly and dramatically the outcome of the program. Assessing the quality of live *Brucella* vaccines is usually based on in vitro criteria, including physico-chemical and microbiological in vitro tests as to purity, dissociation, and determination of pH, humidity and count of viable bacteria [9]. Recently, genetic stability has also been proposed as an additional criterion in assessing of the quality of *Brucella* spp. vaccines [10–12]. Although less frequently, immunogenicity in mouse can also be included in *Brucella* vaccines tests, however not having cutoff points (protection zone) defined for RB51 vaccine [9–11] and as mouse immune system does not accurately represent bovine immune system, it is very difficult to use such data.

Attenuated *B. abortus* strains have demonstrated the best results in the prevention of bovine brucellosis. *B. abortus* live modified vaccines are highly effective in decreasing transmission and production losses caused by brucellosis, but are less effective at preventing infection by field strains [4]. Since, abortion is the key for the brucellosis transmission in cattle, a vaccine that can effectively prevent abortion is able to reduce disease transmission and largely reduce economic losses caused by the disease. Immunization with live modified *B. abortus* vaccines is generally performed in young female calves in a single dose by intramuscular or subcutaneous injection [5]. However, in zones of high prevalence of brucellosis, massive vaccination, including adult cows, is performed.

It is also important to take into account that although cattle are the main target of the vaccination against *B. abortus* within bovine brucellosis control and eradication programs, they are not the unique species infected by this agent. Goats, feral swine, elks, bison and other hosts can also be infected by *B. abortus* and some of them are even able to sustain the disease, being considered an important source of the re-emergency of the disease in cattle [1].

Only a few vaccines have been used massively in cattle immunization against *B. abortus,* S19, RB51, 45/20 and SR82, being S19 and RB51 the most widely used vaccines [4]. However, many *B. abortus* vaccine candidates have been developed, including DNA, subunit, recombinant *B. abortus* and recombinant vector vaccines. All of them are evaluated principally in mouse model [13–46], and with a few exceptions, the majority of these new vaccines, have not been tested in cattle or were not protective in cattle, the target species.

## 3. S19 vaccine

Strain 19 is a live attenuated vaccine and the first *B. abortus* vaccine to be used extensively for bovine brucellosis control [47]. In USA, this vaccine was used for more than five decades from 1941 and is still being used in several other countries [4].

*Brucella abortus* S19 was isolated in 1923 from milk of a Jersey cow by Dr John Buck [48]. This virulent culture was accidentally left out at room temperature for one year and when tested in guinea pigs showed lower virulence compared with previous tests [49]. Subsequently, S19 showed to be highly successful in immunization of calves [48,50]. The efficacy of *B. abortus* S19 was proved by experimental tests in cattle [47,51] and under field conditions [5,52]. Its main characteristics are stable low pathogenicity, relatively high immunogenicity, and moderate antigenicity [53]. Strain 19 is a smooth attenuated *B. abortus* biovar 1 that induces antibody response that cannot be distinguished from the antibody response induced by infection with field strains [4,50]. The lipopolysaccharide (LPS) O-side chain is an immunodominant antigen to which the majority of antibodies resulting from S19 immunization or natural infection are directed [54]. Antibody titers resulting from vaccination may persist for a prolonged period in a small proportion of vaccinated calves: approximately two animals per 100 000 calfhood vaccinated ones [54]. Residual antibody titers increase with the age at which the animal was vaccinated [5], and to address this issue, vaccination is usually performed on young female calves between three and eight months of age [47]. However, vaccination of this age group does not appear to significantly differ in immunity induced [47]. Restriction on age of vaccination, due to the interference in the brucellosis diagnosis, is the main disadvantage of vaccination with S19. This has contributed greatly to their replacement by RB51 vaccine strain, which does not have this problem.

In calves, S19 vaccination can be performed with full dose [2.5 – 12 × 10^10^ colony forming units (CFU)], original dosage used in S19 classical experiments, or with reduced dosage (3–10 × 10^9^ CFU) to minimize residual antibody titers and to prevent occasional persistent vaccinal infection [4]. After calfhood-vaccination, S19 is usually cleared from superficial cervical lymph node within 10 to 12 weeks. Vaccination of adult cattle with S19, low dosage (0.3-3 × 10^9^ CFU), was also successfully employed in infected herds [5,55,56]. S19-adult vaccination was tested as a strategy to be used in infected herds in order to reduce abortions and subsequently brucellosis transmission; however, it was discontinued because vaccination of pregnant animals can cause abortion and mainly because of the persistence of vaccinal antibodies [56,57].

In general, after calfhood vaccination, S19 does not persist in the reproductive tracts of mature heifers and does not cause abortion in these animals [54]. Nonetheless, even with markedly infrequent occurrence, some cattle remain chronically infected and may abort and excrete the vaccine strain in the milk. Another disadvantage of S19 vaccination includes the fact that in some circumstances S19 can cause abortion in pregnant animals [57]. After vaccination of cattle with one, two or three doses prior to breeding age, McDiarmid [51] recovered S19 from 10% of milk samples and 1.5% of samples from cases of abortion. In males, calfhood S19 vaccination usually results in persistent antibody titers, testicular infection, and hence infertility [58]. Furthermore, the vaccination of infected animals with S19 does not cure nor alter the normal course of the disease [47,50].

On the other hand, duration of immunity induced by S19 in cattle vaccinated as calves has proven to be quite long, reaching almost the entire productive lifespan of the animal [47,51]. The immunity in cattle vaccinated between 6 and 8 months of age does not decrease from the first through the fifth pregnancy [47,51]. Moreover, revaccination experiments with S19 and killed *B. abortus* vaccines demonstrated no apparent benefit in cattle-challenge experiments compared with just S19-calfhood immunization [47], despite McDiarmid [51] having observed a small gain from S19 revaccination. Under field conditions 82 to 95% of vaccinated cattle have been shown to have complete protection against infection with virulent strains [50]. However, it has also been demonstrated that the effectiveness of the vaccine decreases proportionally with an increasing dose of bacterial exposure [47,50].

Regarding the immune response triggered by S19 vaccination, most of our knowledge comes from mice studies, which have been shown a strong Th1 immune response with production of IFN-γ and high levels of antigen-specific CD4^+^ and granzyme B-secreting CD8^+^ T-cell responses [32].

Being pathogenic to man, the utilization of S19 requires safety training of the personal involved and the use of personal protection equipment as gloves, long sleeve coats, protection glasses, and N95 masks.

## 4. RB51 vaccine strain

*B. abortus* strain RB51 is a rough rifampicin-resistant strain, which exhibited a lack of expression of the LPS O-side chains (OPS) [59]. RB51 vaccine strain was developed in 1982 by Prof. Gerhardt Schurig’s group and is derived from a virulent smooth *B. abortus* biovar 1 strain 2308 [59]. This is a natural mutant derived by a serial passages on media containing subinibitory concentrations of rifampicin or penicillin and by selecting single colonies with rough morphology [59]. The rough characteristic is stable during in vitro and in vivo passages and does not revert to virulent phenotype [59].

The protection against abortion and infection induced by RB51 vaccination in cattle has been sufficiently demonstrated under experimental conditions [6,7,60,61,64]. Also, the use of RB51 is highly effective under field conditions, in herds with high and low brucellosis prevalence [62,63].

The literature shows that calves vaccinated with RB51 at three, five and seven months of age are protected against infection and abortion [64], as well as heifers vaccinated at age of 10 or 24 months, after challenge with the virulent *B. abortus* 2308 [6,60]. Nevertheless, it has been suggested that under experimental circumstances the vaccination with S19 is slightly (not significant) more efficacious than RB51 [4,64]. After vaccination, RB51 is usually cleared from calf superficial cervical lymph node within 6 to 10 weeks [64]. RB51 is considered more attenuated than S19, based on results of clearance and histologic examination of infected tissues of vaccinated animals.

In general, the recommended dosage for RB51 calfhood vaccination is 1.0 – 3.4 × 10^10^ CFU [4]. Protection against *B. abortus* infection is similar through the suggested dosage, although higher antibody titers and longer persistence of bacteria had been associated with the full dose (3.4 × 10^10^ CFU) [8]. Reduced dosage (1 × 10^9^ CFU), generally recommended for adult animals, also protects against infection and abortion caused by virulent 2308 [61]. Despite RB51 having highly reduced abortifacient characteristics [59,65], it is not completely safe for pregnant cow, mainly when full dose is administrated [66]. However, some results indicate that non-vaccinated cattle and cattle vaccinated with S19 as calves can be safely vaccinated with RB51 (full dose) during the pregnancy [6,67,68]. Furthermore, data indicates that RB51 vaccination is a safe procedure also for males [69].

In addition, as S19 vaccine, RB51 can cause infection in humans especially immunosuppressed individuals [70]. RB51 is resistant to rifampicin, one of the antibiotics of choice in the treatment of human brucellosis, and failure to be detected by routine serological tests are the two most important points one has to be aware during diagnosis and treatment of humans. Therefore, the same protective measures recommended for S19 also applies to RB51 use.

Because of the rough phenotype, RB51 does not induce the production of anti-OPS antibodies in immunized animals, overcoming the serologic problems observed after S19 vaccination [7,59,61]. Consequently, RB51 vaccinated cattle can be easily and accurately differentiated from naturally infected animals, allowing the effective use of the test-and-slaughter and vaccination policies simultaneously. Vaccination with RB51 does not induce antibodies detectable by routine serologic brucellosis diagnostic tests, even after S19 calfhood vaccination and multiple RB51 boosters or use of full dose of RB51 (3.4 × 10^10^ CFU) [7,61,68]. However, RB51-specific antibodies can be detected by dot enzyme-linked immunosorbent assay or ELISA using killed RB51 antigens [71,72], until approximately 12 weeks after vaccination, with the peak occurring four weeks after vaccination or revaccination with decreasing titers after ten weeks [61,73]. Interestingly, S19-vaccinated cattle exhibit higher titers against RB51 antigens in ELISA than animals vaccinated with RB51, probably due to persistence of S19, which may result in high levels of cross-reacting antibodies against RB51 antigens [73].

So far, there are no experiments that evaluated the duration of immunity, but Olsen and Stoffregen [4] suggest that a booster vaccination is required between 4 and 5 years of age to maintain high levels of protection after RB51 calfhood vaccination. Also, RB51 revaccination has been recommended six months and one year after calfhood vaccination in northern Mexico [74]. Nonetheless, findings from blastogenic response of CD4^+^ and CD8^+^ T-cells and the production of IFN-ɣ and IL-4 by the lymphocyte subsets six months after RB51 revaccination indicate that there was no increase or improvement in the immunological response resulting from RB51-revaccination of adult cattle [75]. Even though, RB51 revaccination may still be considered as a tool for increasing herd immunity, since not all animals are completely protected after primary immunization [50]. Furthermore, it has been demonstrated that RB51 induces a strong Th1 cellular immune response with production of IFN-γ and CD8^+^ specific cytotoxic cells, but not IL-4 after vaccination of mice [76].

## 5. 45/20 vaccine

This vaccine is prepared with heat-killed *B. abortus* biovar 1 strain 45/20 combined with oil adjuvant [77]. The 45/20 is a rough *B. abortus,* derived of smooth strain 45/0 after 20 passages through guinea pigs [78]. This bacterin was used in some European Union countries for *B. abortus* control replacing S19, in order to eliminate the problems related to the induction of antibodies interfering in the routine diagnosis of infection [5]. However, data of experimental efficacy and immunologic response are contradictory and mostly show the superiority of vaccination with S19 [79,80]. Furthermore, its use has some drawbacks such as the use of oil adjuvant, needing of repeat vaccination and reversion to smooth strain when used as a live vaccine [5,78]. Furthermore, some studies have also indicated that 45/20 is not completely free of the O-side chain [81], hence this vaccine can induce antibodies detectable by routine serologic tests employed in the diagnosis of bovine brucellosis. The variability in reported protection, along with unpredictable serological effects and the occurrence of reactions at the site of vaccine injection in some animals led to the interruption of the use of 45/20 vaccine.

## 6. SR82 vaccine strain

The SR82 strain is a *B. abortus* biovar 6 live attenuated vaccine used since 1974 by the former Union of Soviet Socialist Republics (USSR) for bovine brucellosis control [82]. This vaccine agglutinates in both rough and smooth anti-sera, but does not induce positive response in brucellosis agglutination tests [82,83]. Moreover, SR82 induced protection level similar to S19, after challenge with virulent *B. abortus*, and it has been shown to be effective under field conditions [82,83]. Currently the SR82 strain is still massively used in the Russian Federation, Azerbaijan, Tejikistan and other countries in the region [82].

## 7. Vaccination with recombinant genes, proteins, vectors and *B. abortus* recombinant mutants

Classically and historically the vaccines used in the bovine brucellosis control are live attenuated vaccines produced from spontaneously attenuated or randomly selected strains. Nonetheless, the numerous advances in genomics, proteomics, recombinant DNA technology and even in vaccinology, allowed the exploration of other tools for the development of safer vaccines, without drawbacks observed in classical vaccines. In this context, several studies aimed to develop, test the efficacy or assess the immulogical responses of the *B. abortus* genetically engineered vaccines (recombinant genes, proteins, vectors and modified *B. abortus* strains) have been performed essentially in mice. However, with a few exceptions the majority of these recombinant vaccines, have not been tested or did not protect cattle, their target species. Moreover, it is important to take into account that recombinant vaccines, especially non-living ones, have limitations regarding economic viability, need for multiple doses and the need for combination of antigens.

### 7.1. DNA vaccines

DNA vaccines offer the possibility of inducing both cellular and humoral responses, expression of antigens is prolonged, they have better stability and do not require refrigeration under storage. Therefore, several antigens have been explored for their value as DNA vaccines against *B. abortus* challenge, providing various levels of protection. DNA vaccines encoding ribosomal *L7/L12,* lumazine synthase (*BLS*), *P39* (a putative periplasmic binding protein), *Omp16* (outer membrane protein) and *BAB1_0278* genes have demonstrated to confer protection against *B. abortus* challenge in mice [22,23,27,42]. Moreover, Cu/Zn superoxide dismutase (Cu-Zn SOD) DNA vaccine induced a protection level similar to the one induced by RB51 [25]. All these genes also proved capable of eliciting a desirable cellular immune response in mice [22,23,32,33,42]. In contrast, plasmid DNA carrying the *BAB1_0263* and bacterioferritin (*BFR*) genes did not induce significant level of protection against challenge with virulent *B. abortus* in mice.

Combined DNA vaccines have also demonstrated their ability to protect better against challenge. DNA vaccines of genes coding for an immundominant *Brucella-*antigen (BCSP31) and promising *Brucella-*antigens (SOD and L7/L12) provided significantly better protection than S19 in mice [32]. This combined DNA vaccine also elicited significantly higher cytotoxic response (granzyme B–producing CD8^+^ T cells) compared to S19-vaccinated mice [32]. Likewise, divalent fusion DNA vaccine encoding *L7/L12* and *Omp16* genes also proved to be effective and able to elicit a strong T-cell proliferative response and induce a large amount of IFN-γ producing T cells [27]. Additionally, data showed that combination of these *B. abortus* genes (*BCSP31, SOD* and *L7/L12*) with *Mycobacterium tuberculosis* (*Ag85B, MPT64*, and *MPT83*) or *Mycobacterium bovis* (*Ag85B, MPT64,* and *MPT83*) genes are very promising for both agents [33,34]. DNA vaccine containing six genes encoding immunodominant antigens from *M. bovis* and *B. abortus* induced protection comparable to S19 and better than Bacillus Calmette-Guerin (BCG) vaccine in cattle, suggesting that this is a highly promising vaccine for both diseases [34]. Combined DNA vaccine containing *M. tuberculosis* and *B. abortus* genes with added IL-12 adjuvant system showed that besides the high level of protection, IL-12 acts as an adjuvant to enhance protective immunity against *M. tuberculosis* and *B. abortus* in challenge mice [33]. Conversely, results suggest that a SOD DNA vaccine fused to IL-2 did not improve protection efficacy [29].

However, despite some of *B. abortus* DNA vaccine candidates have shown very promising results in mice, the need of at least four booster vaccinations to be effective as well as the high cost for use in large animals, make this type of vaccine impractical for cattle, the main target of brucellosis vaccination. Moreover, excluding mouse studies practically no DNA vaccine has been explored in natural hosts.

### 7.2. Subunit vaccines

Many of the same antigens tested as DNA vaccines have also been evaluated as potential antigens for subunit vaccines (L7/L12 ribosomal protein; P39; BLS; Omp16; Cu/Zn SOD) [14,21,24,37,40]. The outer membrane proteins (OMPs) of *B. abortus*, potential immunogenic antigens, have been widely explored as subunit vaccines [37,39,43,45]. Unlipidated recombinant Omp16 and Omp19, and recombinant Omp25 liposome encapsulated gave protection comparable to S19 in vaccinated mice following challenge [37,39,45]. Also, Omp28 subunit vaccine increased resistance against challenge with virulent *B. abortus* but at lower level than live attenuated vaccines [43].

Similarly, flagellar proteins have been screened in search for a subunit vaccine antigen candidate. Five flagellar genes, although *Brucella* spp. are non-motile, (*BAB1_0260* (FlgJ); *BAB2_0122* (FliN); *BAB2_0150*; *BAB2_1086*; *BAB2_1093*) were evaluated for their ability to induce humoral and cell-mediated responses and protect mice against *B. abortus* challenge [84]. Of these, FlgJ and FliN were found to be protective antigens that produced humoral and cell-mediated responses in mice.

Moreover, recombinant proteins of other proven or putative pathogenesis-associated genes such as L7/12, BLS, rSurA and rDnaK induced different levels of protective immunity and cellular immune response in mice against brucellosis [14,24,31]. Whereas, dihydrolipoamide succinyltransferase (rE2o) and cysteine synthase A (rCysK) provided partial protection against *B. abortus* challenge and induced primarily Th2 type of immune response [41,46]. Furthermore, CobB, AsnC and P39 elicit protective immunity similarly to Cu/Zn SOD and S19, which is marked by both humoral and cellular immune responses [21,85]. Also, Cu/Zn SOD recombinant protein (liposomes encapsulated) confers resistance in mice, further increased upon co-immunization with recombinant IL-18 [40]. In contrast, BAB1_0560, BAB1_1108, BAB2_0059 (VirB10), BAB2_0191, BAB2_0423 (GntR) and BRF protein vaccines did not induce protective immune response [21,85].

The potential use of *B. abortus* subunit vaccines under field conditions is very limited, although some encouraging results showed. The requirement of multiple boosters, adjuvants and combination of several antigens makes it economically unsuitable for cattle. Moreover, it is important to consider that the response observed in mice may not reflect the protection achieved in natural hosts after vaccination. Furthermore, to generate a strong and protective immune response that can mimic the natural infection from a combination of few proteins of the pathogen is a hard and complex challenge.

### 7.3. Vector vaccines

Alternatively, genes encoding immunodominant *B. abortus* antigens can be introduced into attenuated viruses or bacteria that serve as vector vaccines. *B. abortus* genes have been successfully expressed in viruses (Semliki Forest virus and Vaccinia virus) and bacteria (*Escherichia coli, Ochrobactrum anthropi, Lactococcus lactis*, *Salmonella enterica* subsp *enterica* serovar Typhimurium and *B. abortus*) [16,20,26,28,35,38,44,86]. *Escherichia coli, O. anthropi* (plus unmethylated CpG motifs) and *L. lactis* expressing Cu/Zn SOD antigen of *B. abortus* were able to elicit a Th1 immune response and to protect mice following challenge with virulent *B. abortus* [20,28,44,86]. Likewise, Semliki Forest virus-based vector carrying RNA encoding *Brucella* translation initiation factor 3 (IF3) showed a significant level of protection against a challenge with *B. abortus* 2308 in mice [35]. L7/L12 protein carried by *S. enterica* serovar typhimurium but not by Vaccinia virus conferred protective efficacy and immunogenicity [26,38]. Also, vaccinia virus carrying 18-kDa OMP of *B. abortus* were not able to protect mice against a challenge with the virulent strain *B. abortus* 2308 [87].

The expression of *B. abortus* antigens on viral or bacterial vectors is a superior alternative to DNA and subunit vaccines, as it closely mimics the natural infection, allowing the modulation of the host immune response and the multiplication of the initial number of antigen copies within the host. However, despite not having some of the inconveniences observed in non-living vaccines, as multiple doses, need for adjuvant and high cost, other organisms expressing *B. abortus* proteins still need the perfect grouping of antigens, expressed in high amount to be effective. The amount of foreign protein expressed by the carrier organism needs to be able to promote a specific protection. Moreover, the use of viral platform implies in small chance that the vector DNA is integrated into the genome of the host cell. In addition, although promising most of these above vaccines have failed or have not been tested in cattle, the target species, so no conclusion could be drawn at this time.

### *7.4. B. abortus* recombinant mutants

Another focus of research for new vaccines to protect against *B. abortus* infection has been the construction of RB51 recombinant mutants, which retain the rough phenotype and attenuation but have improved characteristics such as immunogenicity and protection against a challenge [17–19]. Hence, some studies have shown that the complementation of RB51 with a functional *wbo*A gene (RB51*Wbo*A), which lead to the expression of O-side chain in its cytoplasm, or the overexpression of Cu/Zn SOD protein (RB51SOD) results in significant enhancement of the vaccine efficacy against challenge with virulent *B. abortus* in mice [17,18]. Furthermore, the combination of these two genes in a single RB51 strain (RB51SOD/*wbo*A) also significantly increased the protective ability of this RB51 recombinant vaccine in mice and did not alter its desirable characteristics [19]. Nonetheless, this RB51-recombinant strain was not as effective as the parental RB51 strain in calfhood vaccination of bison after challenge with 2308 [36].

Besides RB51, *B. abortus* strain 2308 has also been tested as recombinant mutant vaccine; the deletion of the gene *znuA,* important protein for survival and normal growth under low Zn^2+^ concentrations, generated a mutant capable of conferring protection similar to S19 or RB51 against challenge with parental 2308 in mice [30]. Experiments in natural hosts, cattle, showed that the double gene deletion (*htrA cycL*) PHE1 was attenuated in the bovine host when compared to the virulent parental 2308 [88]. However, due the absence of a standard challenge study using this potential vaccine, the meaning of such data is unclear. Recombinant mutants based on deletion of ABC transporter ATPase (*BAB1_0542*) or phosphoglycerate kinase (*pgk*) gene of *B. abortus* 2308 also showed protection against challenge with virulent strain in mice and the critical role of these genes for full bacterial virulence [89,90]. In addition to the virulence attenuation, it is desired that these *B. abortus* mutants also show no interference with the diagnostics tests, hence genes associated to the smooth phenotype have been explored in the generation of deleted vaccines. Rough mutant generated by *wboA* gene deletion of S19 protected mice against challenge with 2308 and did not induce abortion in pregnant sheep, showing promising results to be explored in the future development of rough vaccines [91].

The improvement of the existing *B. abortus* vaccines or the creation of new attenuated vaccines by deletion or complementation of some genes seems to be the most promising direction to find a safer and more efficient substitute for the known *B. abortus* vaccines. Modified live vaccines are highly effective in comparison to killed vaccines. This is most likely due to strong and protective cellular immune response induced by live vaccines [2,3]. The use of this platform avoids the main disadvantages related to the non-living vaccines, as multiple delivery, low immunogenicity, need for adjuvants and high cost. Furthermore, *B. abortus* strains, even if genetically modified, can colonize, be immunogenic, and therefore perfectly simulate the natural infection. They are able to multiply within animals for a short period expressing in vivo protective antigens. The major advantage of this approach over the use of vectors is that recombinant mutants share most of proteins with *B. abortus* field strains, whereas carrier organisms are able of expressing only few *Brucella* antigens. However, a real concern on *B. abortus* mutant strains is the presence of antibiotic selection marker. The antibiotic marker is used in the screening of transformed clones, but it is not desirable in the final vaccine due to the potential of spreading antibiotic resistance genes. Options, as an RB51 leucine auxotroph, have been explored to avoid this issue [92]. Additionally, so far, there is no data available to exclude the possibility that these live mutants will not have similar safety and diagnostic issues as live strains, especially if made from smooth strains. Also, to move forwards in the control of bovine brucellosis, these recombinant mutants must be evaluated in cattle and other target animal species. There is still a worldwide need for a vaccine that is safe and highly efficacious in natural hosts, since the transmission of disease occurs from cattle to people. The results obtained in mice, although favorable for some vaccines, have to be interpreted according to their limitations, as they cannot be directly extrapolated to cattle.

On another point of view about the usefulness of *B. abortus* mutant as vaccines, S19 and RB51, the widely used *B. abortus* vaccines, has been investigated as potential vectors for heterologous protein expression, mainly using protective antigens important for other diseases of veterinary interest [93–96]. In this context, multivalent recombinant RB51 vaccines expressing *Neospora caninum* or *M. bovis* proteins have been shown to induce antigen specific immune response to heterologous antigens and, in the case of *N. caninum,* was also achieved significant level of protection in mice [93,94]. Likewise, S19 carrying the genes encoding for the heterologous antigens of *Babebia bovis* or *M. bovis* demonstrated successful specific cellular immune response to recombinant proteins in mouse model [95,96]. These above bivalent live modified *B. abortus* candidate vaccines need further evaluation as to their ability to induce protective immune response as well lack of interference in the diagnostic tests.

## 8. Other *B. abortus* potential vaccines

Besides *B. abortus* recombinants vaccines, also vaccines based on outer membrane vesicles (OMVs) has been exploited as an acellular alternative to live vaccines [97]. OMVs are bilayer membrane vesicles release by Gram-negative and Gram-positive bacteria, which have been associated to many processes such as release of virulent factors, DNA transfer, regulation of host immune response and survival in the host cell [98]. *B. abortus* OMVs are mainly composed for outer membrane proteins (Omps) and have been associated with modulation of host immune response by inhibition of TNF-α and IL-8 response, inhibition of IFN-γ induced expression of MHC class II molecules on human monocytes and increase in expression of adhesion molecules [97–99]. A *Brucella melitensis* vaccine based on OMVs has been tested and showed promising results in BALB/c mice [100]. Furthermore, it is already available a vaccine based on OMVs, against meningococcus serogroup B (*Neisseria meningitides*) in some countries as Cuba, Norway and New Zealand [97]. Therefore, it is possible to speculate that an OMV vaccine against *B. abortus* has a great potential to be considered as part of the continuous efforts to reach safer and more effective *B. abortus* vaccine. Nonetheless, due to its high cost and laborious production, OMV is a more suitable approach for human vaccines, being impracticable for cattle bearing in mind the technologies currently available.

## 9. Conclusions

Vaccination is a determinant strategy for brucellosis control and eradication programs, therefore it has been the target of innumerous studies over decades. Nowadays, some effective vaccines are available to control the disease in cattle. S19 and RB51 are the officially approved *B. abortus* vaccine strains more widely and successfully used to prevent bovine brucellosis worldwide. However, due to some side effects shown by these current vaccines, plus the advances in recombinant DNA technology and the lack of a vaccine for humans, there is an on going extensive efforts focused on the development of new and better vaccines. Engineered vaccines have the potential to be the future of the bovine and human brucellosis control, but many studies are still needed to develop a better vaccine than the current vaccines in terms of safety, efficacy and other desirable characteristics. Moreover, it is important to consider that, mainly non-living recombinant vaccines, also present important issues, as the requirement of multiple boosters, adjuvants, and optimal combination of antigens, besides usually inducing poor cellular immune response. In addition, although the excellent results observed for some recombinant vaccines in mice, very few of these candidate vaccines have been evaluated in cattle. The recent studies indicate that the future of a new *B. abortus* vaccine will be the construction of directed mutants, which exclude the drawbacks and simultaneously increase immunogenic characteristics presented by S19 and RB51. Furthermore, concerning the immune response induced after S19 and RB51 vaccination in cattle, as well as after RB51 revaccination, very little is understood. Efforts to find out the principal characteristics of the immune response triggered in cattle by the two most used and successful *B. abortus* vaccine strains are essential to try to establish an ideal vaccine. The definition of immune markers correlated with protection, by mathematical modeling or evaluation of the immune response in vaccine - challenge studies - would be very helpful in the screening of *B. abortus* candidate vaccines.

## 10. Abbreviations

Th1: Type 1 T helper
LPS: Lipopolysaccharide
CFU: Colony forming units
OPS: O-side chains
OMP: Outer membrane protein
Cu-Zn SOD: Cu/Zn superoxide dismutase
BFR: Bacterioferritin
BCG: Bacillus Calmette-Guerin
rCysK: Cysteine synthase A
